# Editorial: The individual and joint contributions of molecular and environmental factors on gene expression and psychopathology development

**DOI:** 10.3389/fpsyt.2025.1599378

**Published:** 2025-04-10

**Authors:** Gabriela Ariadna Martínez-Levy, Diego Luiz Rovaris, Rodrigo G. Arzate-Mejía, Jill A. Rabinowitz

**Affiliations:** ^1^ Genetic Department, National Institute of Psychiatry, Mexico City, Mexico; ^2^ Laboratory of Physiological Genomics of Mental Health (PhysioGen Lab), Institute of Biomedical Sciences, University of Sao Paulo, Sao Paulo, Brazil; ^3^ Laboratory of Neuroepigenetics, Brain Research Institute, Medical Faculty of the University of Zurich and Institute of Neurosciences, Zurich, Switzerland; ^4^ Department of Psychiatry, Robert Wood Johnson Medical School, Rutgers University, Piscataway, NJ, United States

**Keywords:** psychopathology, molecular factors, environmental factors, polygenic score (PGS), epigenetics

After decades of psychiatric research, it is now widely recognized that psychopathology is a product of complex interactions between biological and environmental factors. Understanding the role of early life stress exposures and genetic predispositions on the development of psychiatric disorders is a topic that remains an area of ongoing investigation. Many questions remain about how these interactions drive epigenetic changes that may exacerbate or attenuate risk for mental health conditions. To address these gaps, novel approaches have been developed to study the contributions of genetic, biological, and contextual factors in shaping responses to stress and mental health outcomes across the life course.

With this in mind, we invited researchers to submit manuscripts focused on applying or developing novel methods aimed at elucidating genetic and environmental influences on psychopathology risk or resilience. The studies presented in this Research Topic offer insights into gene-by-environment interplay, deepening our understanding of mental health conditions and guiding future research with potential clinical implications. Notably, the studies included in this Research Topic examine populations historically underrepresented in psychiatric genetics research, addressing a critical gap.

In a pilot study, Holliday et al. explored the potential impact of chronic stress and childhood trauma on salivary microRNAs, important regulators of gene expression and post-transcription processes. Using a college-aged sample of African American women (N = 12), the authors found that individuals who experienced various levels of chronic stress and adversity evidenced different microRNA levels, specifically miR-19b, miR-187, miR-34a, and miR-135-3p. These findings highlight these microRNAs as biological markers that may be uniquely altered based on high chronic stress and adversity exposure. Given the small sample size and exploratory nature of the study, replication in larger samples is needed.

Ota et al. examined the association between the expression of 78 candidate genes previously linked to psychopathology with life adversity, baseline levels and change in internalizing and externalizing symptoms. The sample included 298 children drawn from the Brazilian High-Risk Cohort Study (BHRCS) who were assessed at baseline (M age = 9.87 years, SD = 1.81) and follow-up (M age = 13.14 years, SD = 1.82). At baseline, the expression of *DENND11* and *PRRC1* was negatively associated with environmental deprivation, whereas the *USP38* gene was negatively linked to externalizing symptoms. At follow-up, *NR3C1*, *HSPBP1*, *SIN3A*, *SMAD4* and *CRLF3* were negatively correlated with internalizing symptoms, whereas the *FAR1* gene was positively associated with internalizing behaviors. No candidate genes were linked to changes in psychopathology or reported adversity. These results highlight the relevance of distinct gene expression profiles as early markers of susceptibility to psychopathology symptoms among Brazilian youth.

Another manuscript led by Elam et al. examined whether life adversity and parental acceptance moderated the relationship between polygenic scores of epigenetic ageing (i.e., GrimAge) and depression trajectories during early adolescence. Participants were drawn from the Adolescent Brain Cognitive Development (ABCD) study and included European American (N= 6,043), African American (N=1,640), and Latino (N=2,283) youth assessed at ages 10-11 and followed annually through ages 12-13 years. The authors found that in European American and African American youth, adverse life events -but not parental acceptance-moderated the effect of GrimAge PGS with depression over time such that higher levels of adversity were associated with an increasing depression trajectory among youth higher in GrimAge PGS. These findings support the diathesis-stress model and suggest that the combination of adversity and genetic risk for accelerated epigenetic ageing is associated with increases in depression among European and African American youth.

Additionally, Su et al. examined whether family processes moderated or mediated the relationship between externalizing PGS and externalizing behavioral trajectories across development. Participants were drawn from the ABCD study and included European American (N=5,907), African American (N= 1,694) and Latino American (N=2,117) youth assessed annually from ages 9/10 to ages 12/13. PGS were created based on a genome-wide association study (GWAS) conducted on externalizing behaviors in adults and childhood aggression. Higher PGS for externalizing behaviors based on adult and childhood GWAS on these phenotypes predicted membership in high externalizing behavior trajectories, highlighting the stability of genetic influences on the expression of these behaviors. Findings also revealed evidence of mediation via family processes. For example, among youth of European ancestry, greater PGS for externalizing behaviors based on GWAS conducted in children and adults was associated with higher family conflict, which in turn, was associated with a greater likelihood of membership in high externalizing symptom trajectories. These results highlight the importance of family conflict as an important environmental factor and mechanism that increases the risk for externalizing behaviors over time among youth genetically vulnerable to experiencing these symptoms.

Lastly, this Research Topic includes a study conducted by Tendilla-Beltran et al. that examined the role of hypothalamic-pituitary-adrenal axis dysregulation assessed via zinc pituitary function in relation to suicide risk. The authors analyzed pituitary postmortem samples of 14 suicide completers and nine non-suicidal subjects for zinc homeostasis (fundamental for hormone synthesis) and the presence of microadenomas (small benign tumors in the pituitary). The authors identified disrupted zinc compartmentalization and a higher prevalence of microadenomas in suicide completers compared to controls. These findings suggest novel pathophysiological mechanisms linking endocrine dysfunction in the stress-response system to suicide vulnerability.

In summary, the articles presented in this Research Topic expand our knowledge of the interplay between biological and environmental factors in psychopathology. Through collaborations across disciplines, such as molecular genetics, epigenetics, neuroendocrinology, and psychiatry, we move closer to identifying biomarkers associated with mental health impairments and improving intervention strategies aimed at promoting long-term health and well-being. The inclusion of genetically-informed psychiatric studies that consider ancestrally diverse populations, such as the manuscripts presented herein, has the potential to reduce disparities in knowledge regarding the mental health of underrepresented populations and foster more inclusive and equitable applications of precision medicine initiatives.

